# Imperatorin Ameliorates the Aging-Associated Porcine Oocyte Meiotic Spindle Defects by Reducing Oxidative Stress and Protecting Mitochondrial Function

**DOI:** 10.3389/fcell.2020.592433

**Published:** 2020-12-21

**Authors:** Dan Luo, Jia-bao Zhang, Sheng-peng Li, Wen Liu, Xue-rui Yao, Hao Guo, Zhe-long Jin, Yong-xun Jin, Bao Yuan, Hao Jiang, Nam-Hyung Kim

**Affiliations:** ^1^Department of Laboratory Animals, Jilin Provincial Key Laboratory of Animal Model, Jilin University, Changchun, China; ^2^Department of Animal Science, Chungbuk National University, Cheongju, South Korea; ^3^Department of Laboratory Animals, Southern Medical University, Guangzhou, China; ^4^School of Biotechnology and Healthcare, Wuyi University, Jiangmen, China

**Keywords:** imperatorin, porcine oocyte, aging, oxidative stress, mitochondrial function, autophagy

## Abstract

Imperatorin (IMP) exhibits a variety of pharmacological properties, including antioxidant, anti-inflammatory, antibacterial, anti-cancer, and anti-hypertension activities. However, its effects on animal reproduction systems, especially oocyte development, maturation, and aging are not yet clear. In this study, the effects of IMP on oocyte development and aging as well as the underlying molecular mechanisms were explored. Oocytes were cultured for an additional 24 h for aging. Results revealed that the blastocyst formation and hatching rates of embryos, which were parthenogenetically activated aged oocytes, were significantly increased with IMP treatment (40 μM). Simultaneously, well-distributed cortical granules but no significant difference in zona pellucida hardness were observed after IMP treatment. During this stage, intracellular reactive oxygen species, apoptosis, and autophagy levels were decreased, while mitochondrial membrane potential, glutathione level, and activity of superoxide dismutase and catalase were increased. IMP-treated aged oocytes also showed significantly higher expression of MOS, CCNB1, BMP15, and GDF9 than non-IMP-treated aged oocytes although their levels were still lower than those in the fresh oocytes. These results suggest that IMP can effectively ameliorate the quality of aged porcine oocytes by reducing oxidative stress and protecting mitochondrial function.

## Introduction

As an animal ages, its oocyte quality reduces, which is a major cause of aging-related decline in female fertility (Liu and Keefe, 2002; Eichenlaub-Ritter et al., 2004). Simultaneously, after ovulation, porcine, and bovine oocytes are arrested at metaphase II (MII) stage, ready for fertilization. If fertilization is not successful at a certain stage, MII oocytes undergo a process known as “postovulatory aging” (Lord and Aitken, 2013; Lord et al., 2013; May-Panloup et al., 2016). Aging oocytes exhibit certain biological abnormalities, including zona hardening, cortical granule exocytosis, meiotic abnormalities, mitochondrial dysfunction, and decreased maturation-promoting factor (MPF) expression and ATP production (Wang T. et al., 2017). Aged oocytes often lead to fertilization failure and subsequent embryo developmental arrest, which causing losses in agricultural production (Miao et al., 2009). In animal reproduction, postovulatory oocyte aging leads to fertilization failure, poor embryonic development, increased abortion rate, and decreased offspring longevity (Tarín et al., 2002; Warburton, 2005). Although animal assisted reproductive technology (ART) can alleviate the negative effects of aging on oocytes, preventing aging, and selecting good quality oocytes are crucial for pre-implantation embryo development (Swain and Pool, 2008). Therefore, it is important to develop strategies to delay oocyte aging for ART and animal husbandry production.

Although the molecular signaling pathways involved in cell aging have not been well explored, reactive oxygen species (ROS) has been reported as a potential candidate inducer (Harman, 1988). ROS is mainly derived from the mitochondria and continuously enriched during aging (Goud et al., 2008), which results in impairment of mitochondrial function (Babayev et al., 2016) and reduced ATP production (Koyama et al., 2014), thus triggering oxidative stress and early apoptosis (Lord et al., 2015). In oocytes, excessive oxygen free radicals and mitochondria damage disrupt the relatively stable microenvironment, leading to aging (Ott et al., 2007). However, previous studies have suggested that many natural and synthetic chemicals, depending on their function of reducing ROS-induced apoptosis, mitochondrial damage, autophagy, and abnormal spindle formation (Lee et al., 2013; Ben-Meir et al., 2015; Wang H. et al., 2017), can reduce or relieve oocyte aging both *in vivo* and *in vitro*. Therefore, identifying more compounds and exploring their underlying mechanism are needed for effective inhibition of oocyte aging *in vivo* and *in vitro*.

Imperatorin (IMP), known as {9-[(3-methyl-2-buten-1-yl)oxy]-7H-furo(3,2-g)}, is a naturally occurring furanocoumarin derivative that is mainly distributed in citrus fruits (*Citrus limonum*), umbelliferous vegetables (*Foeniculi Fructus*), and some herbal medicines (*Angelica dahurica* and *Angelica archangelica*). It exhibits various pharmacological properties, including anti-cancer (Rahman et al., 2015), neuroprotective (Steinthor and Sigmundur, 2013), anti-inflammatory (Abad et al., 2010), anti-hypertension (Yan et al., 2010), and antibacterial activities (Rosselli et al., 2007). In recent years, IMP has received increasing attention due to its antioxidant effects. IMP has been shown to regulate the expression of superoxide dismutase, xanthine oxidase, and nicotinamide adenine dinucleotide phosphate oxidase (Cao et al., 2013) and reduce the production of ROS (Budzynska et al., 2015), as well as protect mitochondrial function (Ahmad et al., 2019). More importantly, our previous research showed that 40 μM IMP can improve porcine early embryonic development by reducing ROS production (Luo et al., 2020). However, whether IMP exerts protective effects on aged oocytes remains unknown.

In this study, we hypothesized that IMP maintains porcine oocyte quality during the process of aging. We investigated the effect of IMP on blastocyst formation rate, hatching rate, distribution of cortical granules, and hardness of zona pellucida (ZP) in parthenogenetically activated aged oocytes. Next, antioxidant capacity, mitochondrial membrane potential (MMP), ATP production, and autophagy levels were evaluated to explore the underlying mechanism. Our findings will contribute to the understanding about the molecular mechanism of oocyte quality control and providing new insights into the oocyte maturation, early development, preventing oocyte aging, and improving animal reproduction.

## Materials and Methods

### Ethics Statement

All experiments were conducted at the Experimental Animal Center of Jilin University in accordance with the Institutional Animal Care and Use Committee of Jilin University (IACUC-ID-201802070).

### Regents and Chemicals

All chemicals and reagents used in this study were purchased from Sigma-Aldrich (St. Louis, MO, United States) unless otherwise indicated.

### *In vitro* Maturation and Aging of Porcine Oocytes

Prepubertal porcine ovaries were obtained from a local slaughterhouse and transported to the laboratory in a sterile saline solution supplemented with 75 μg/mL penicillin G and 50 μg/mL streptomycin sulfate within 2 h at 30–35°C. Cumulus-oocyte complex (COC) was aspirated from a follicle (diameter of 3–6 mm) using a 10 mL syringe with an 18-gauge needle. COCs were washed three times in Tyrode’s lactate-4-(2-hydroxyethyl)-1-piperazineethanesulfonic acid (TL-HEPES) supplemented with 0.1% polyvinyl alcohol (PVA, w/v) and 0.05 mg/mL gentamycin. Only oocytes with a minimum of three layers of cumulus cells were selected. Next, about 50 COCs were matured in 500 μL *in vitro* maturation (IVM) medium [M199 with 10 ng/mL epidermal growth factor, 1 μg/mL insulin, 75 μg/mL kanamycin, 0.91 mM sodium pyruvate, 10% prepubertal porcine follicular fluid (Grupen et al., 2003), 0.5 μg/mL follicle-stimulating hormone, and 0.5 μg/mL luteinizing hormone] with mineral oil for 44 h at 38.5°C in a humidified atmosphere of 5% CO_2_ and 95% air.

For *in vitro* oocyte aging, cumulus cells were removed from the COCs by pipetting in TL-HEPES supplemented with 1 mg/mL hyaluronidase after porcine oocyte maturation. Only denuded oocytes with first polar bodies (fresh group) were used for subsequent experiments. Oocytes were then cultured in mineral oil-covered fresh IVM medium with (IMP-aged group) or without (aged group) 40 μM IMP (Selleck, Shanghai, China) based on our previous research (Luo et al., 2020) for an additional 24 h at 38.5°C in a humidified atmosphere of 5% CO_2_ and 95% air.

### Parthenogenetic Activation and Embryo *in vitro* Culture

Following aging, the oocytes were parthenogenetically activated using two direct-current pulses of 120 V for 60 μs in 297 mM mannitol containing 0.5 mM HEPES, 0.1 mM CaCl_2_, 0.05 mM MgSO_4_, and 0.01% PVA. The activated oocytes were then cultured in bicarbonate-buffered porcine zygote medium-5 (PZM-5) (Yoshioka et al., 2008) containing 4 mg/mL bovine serum albumin [*in vitro* Culture (IVC) medium] and 7.5 mg/mL cytochalasin B for 3 h to suppress the extrusion of the pseudo-second-polar body. After careful washing the oocytes with PZM-5 three times, approximately 50 parthenogenetically activated oocytes were transferred into 4-well plates containing 500 μL of IVC medium and cultured at 38.5°C in a humidified atmosphere of 5% CO_2_ and 95% air. The blastocyst rates (number of blastocysts versus number of cleaved embryos) and hatching rates (number of hatched blastocysts versus number of cleaved embryos) were detected on days 7.

### ZP Hardness Assay

The assay for ZP hardening was carried out according to a previously described method with some modifications (Coy et al., 2008). Briefly, denuded porcine oocytes were transferred into PBS-PVA, washed by pipetting, and transferred into 50 μL drop of 0.5% (w/v) pronase (from *Streptomyces griseus*) solution in PBS-PVA. Zonae pellucidae were continuously observed for dissolution under an inverted microscope at 37°C. Oocytes were observed every 30 s initially, and when the ZP becomes noticeably thinner, they were observed every 5 s at microscope until all the ZP dissolved. The dissolution time of the zona of each oocyte was registered as the time interval between placement of the samples in pronase solution and that when the zona was no longer visible.

### Cortical Granule Distribution Assay

For cortical granule staining, the ZP of oocytes was removed by brief incubation in acidic Tyrode’s solution. The oocytes were fixed in 3.7% paraformaldehyde in PBS-PVA for 30 min and then blocked in PBS-PVA containing 0.3% BSA and 100 mM glycine. Next, the oocytes were permeabilized in PBS-PVA containing 0.1% Triton X-100 for 5 min. After three washes with PBS-PVA, cortical granules were labeled with 10 μg/mL of Alexa Fluor 488-conjugated wheat germ agglutinin (Invitrogen, Grand Island, NY, United States) in PBS-PVA for 30 min in the dark. Finally, oocytes were washed three times, mounted on glass slides, and observed under a Zeiss LSM 510 confocal microscope (Carl Zeiss, Jena, Germany).

### Annexin-V Staining

An Annexin-V staining kit was used for the detection of early-apoptosis (Vazyme, Nanjing, China). The oocytes were incubated at room temperature in the dark for 10 min with 100 ml of binding buffer containing 10 ml of Annexin-V-EGFP. After three times washes in PBS-PVA, the oocytes were mounted on glass slides covered with cover slips. Then, oocytes were determined immediately with the confocal microscope (Carl Zeiss).

### Assays for Intracellular ROS and GSH Levels

To measure intracellular ROS and GSH levels, oocytes were incubated in PBS-PVA containing 10 μM 2′,7′-dichlorodihydrofluorescein diacetate (DCFH, for ROS level measurement; Invitrogen) or 10 μM 4-chloromethyl-6,8-difluoro-7-hydroxycoumarin (CMF_2_HC, for GSH level measurement; Invitrogen) for 30 min. After washing the oocytes three times in PBS-PVA, images were captured using a fluorescence microscope (Nikon, Tokyo, Japan) and ImageJ software (NIH, Bethesda, MD, United States) was used to analyze the fluorescence intensities.

### Superoxide, Superoxide Dismutase, and Catalase Assay

The superoxide level, superoxide dismutase (SOD) activity, and catalase (CAT) activity were assayed by Superoxide Assay Kit (Beyotime, Shanghai, China, #S0060), Total Superoxide Dismutase Assay Kit with WST-8 (Beyotime, #S101S), and Catalase Assay Kit (Beyotime, #S0051) according to the manufacturer’s instructions, respectively. Briefly, before measurement, standard reaction solutions and curve were prepared according to the manufacturer’s instructions. Then, 150 oocytes were dissolved with related lysed buffer and incubated with reaction buffer for 30 min. Next, the absorption value was measured with a microplate reader (Tecan, Mannedorf, Switzerland). The superoxide level and activity of SOD and CAT were calculated based on the absorption value and standard curve.

### Determination of Mitochondria Distribution

To assess mitochondria distribution, after denudation treatment, denuded and washed oocytes were incubated in TCM-199 medium (Invitrogen) for 10 min to acclimatize. Next, denuded oocytes were incubated in TCM-199 medium containing 500 nM MitoTracker Red CMXRos (Cat#M7512; Invitrogen) for 30 min at 38.5°C, and subsequently fixed in PBS-PVA containing 3.7% paraformaldehyde for 30 min at room temperature. After washing three times in PBS-PVA, the oocytes were mounted on glass slides and observed under a Zeiss LSM 510 confocal microscope (Carl Zeiss). ImageJ software were used to analyze the fluorescence intensities.

### MMP (ΔΨ) Assay

To determine the MMP, oocytes were incubated in IVM medium containing 2 μM 5,5′,6,6′-tetrachloro-1,1′,3,3′-tetraethylbenzimidazolylcarbocyanine iodide dye (JC-1; Beyotime) for 2 h with or without IMP treatment at 38.5°C. After washing the oocytes three times in PBS-PVA, images were captured using a fluorescence microscope (Nikon) and ImageJ software was used to analyze the fluorescence intensities. The average MMP of oocytes was calculated as the ratio of red fluorescence intensity to green fluorescence intensity.

### Determination of ATP Levels

The ATP levels in oocytes were measured using an ATP Determination Kit (Cat#A22066; Invitrogen) and a luminometer (CentroPro LB 962; Berthold Technologies, Bad Wildbad, Germany) according to the manufacturer’s instructions, as previous described (Niu et al., 2019). Briefly, oocytes were collected into a 0.2 mL centrifuge tube containing 30 μL lysis buffer (20 mM Tris, 0.9% Nonidet-40, and 0.9% Tween 20) and lysed by ultrasonic shock. Before measurement, standard reaction solutions were prepared according to the manufacturer’s instructions and placed on ice in the dark. The sample lysis solution (5 μL) was then added to a 96-well plate and equilibrated for 10 s. Subsequently, 200 μL standard reaction solution was added into each well, and the light signal was integrated for 10 s after a delay of 2 s. The light intensity in the control group was arbitrarily set as 1, and the light intensity in the treatment group was then measured with a microplate reader (Tecan) and expressed as values relative to the control group.

### Immunofluorescence Staining

Oocytes were fixed in PBS-PVA containing 3.7% paraformaldehyde for 30 min and permeabilized by incubation in 0.3% Triton X-100 for 15 min at room temperature. Oocytes were then blocked in PBS-PVA containing 1% BSA for 1 h. Next, oocytes were incubated with primary anti-LC3B antibody (Abcam, Cambridge, MA, United States; #ab48394) or anti-α-tubulin-FITC antibody (1:200; Abcam; #ab64503) overnight at 4°C. After washing three times in PBS-PVA, the oocytes were incubated with a secondary antibody (Abcam; #ab150073, for LC3B staining) for 1 h at room temperature. Then, DNA was stained with 1 μg/mL Hoechst 33342 for 15 min. Finally, the oocytes were mounted onto glass slides and the fluorescence intensities were examined using a confocal laser scanning microscope (Carl Zeiss). Autophagy levels in the embryos were measured by counting the number of LC3B dots.

### Quantitative RT-PCR Analysis

mRNA was extracted from 150 oocytes using the Dynabeads^TM^ mRNA DIRECT^TM^ Purification Kit (Invitrogen) according to the manufacturer’s instructions. A fast reverse transcription kit (TIANGEN, Beijing, China) was used for the synthesis of cDNA by reverse transcription. Each 20 μL quantitative RT-PCR (qRT-PCR) mixed system included 8 μL of deionized water, 10 μL of SuperReal PreMix Plus (TIANGEN), 1 μL of cDNA, and 0.5 μL each of forward and reverse primers (10 mM). Gene expression was quantified with the Mastercycler ep realplex system (Eppendorf, Hamburg, Germany) using the 2^–Δ^ ^Δ^ ^*Ct*^ method with GAPDH as the internal standard. The PCR conditions were as follows: 95°C for 3 min; 45 cycles at 95°C for 15 s, 60°C for 30 s, and 72°C for 30 s. All primers used are listed in Supplementary Table 1.

### Western Blotting

300 oocytes were collected and placed in lysis and loading buffer (40% ddH_2_O, 12.5% 0.5 M Tris–HCl, 10% glycerol, 2% SDS, 20% β-mercaptoethanol, and trace bromophenol blue) for 10 min at 95°C. Next, total protein was separated on 15% polyacrylamide gels containing 0.1% SDS and transferred onto polyvinylidene fluoride membranes (Millipore, Billerica, MA, United States). After blocking with 5% BSA diluted in Tris-buffered saline with 0.1% Tween 20 (TBST) for 1 h at 37°C, the membranes were incubated overnight at 4°C with primary antibodies against LC3B, cleaved-Caspase 3 (Abcam; #ab49822), and GAPDH (Abcam; #ab9484). After washing three times in TBST for 10 min each, membranes were incubated at room temperature for 1 h with horseradish peroxidase-conjugated goat anti-rabbit IgG (Abcam; #ab205718, for LC3B and cleaved-Caspase 3) and goat anti-mouse IgG (Abcam; #ab205719, for GAPDH). Blots were visualized using a charge-coupled device camera and UviSoft software (Uvitec, Cambridge, United Kingdom).

### Statistical Analysis

All statistical analyses were performed using SPSS software, version 22.0 (IBM Corporation, Chicago, IL, United States). Data from two groups were compared using the Student’s *t*-test. Differences between three or more groups were analyzed using one-way analysis of variance (ANOVA) with the Tukey–Kramer test. All data are presented as the mean ± standard deviation (SD). The total numbers of oocytes or embryos (*N*) used in each group and independent repeat times (*R*) of experiments are shown in the figure notes. Black dots represent the measurements in each group. *P* < 0.05 and *P* < 0.01 were considered statistically significant.

## Results

### IMP Supplementation Maintained the Quality of Aged Oocytes and Enhanced the Developmental Competence of Parthenogenetic Embryos Derived From Aged Oocytes

**FIGURE 1:**
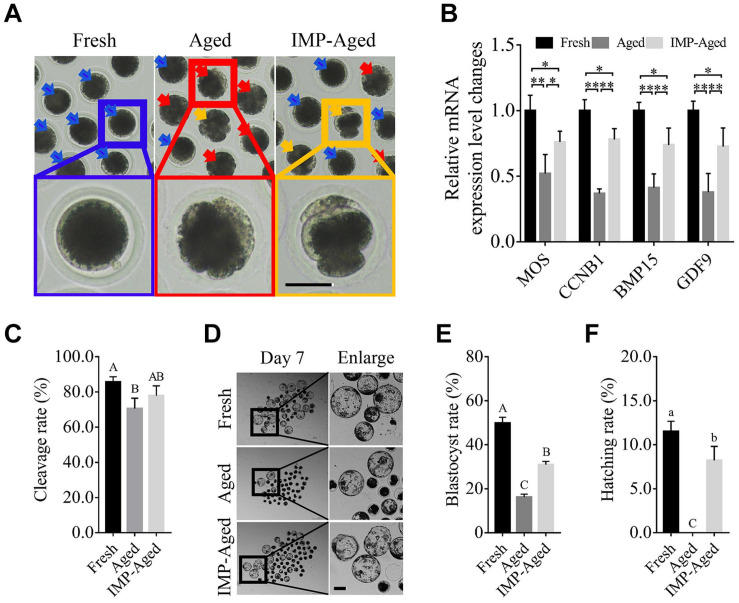
IMP supplementation enhanced the development of porcine parthenogenetic embryos derived from aged oocytes. **(A)** Representative images of oocytes in fresh, aged, and IMP-treated aged groups. Normal/abnormal cytoplasm/spontaneous parthenogenetic activated oocytes were labeled with blue/red/orange arrows. Scale bar = 50 μm. **(B)** Differential gene expression in fresh, aged, and IMP-treated aged oocytes. Gene expression was detected in oocytes after 24 h of aging. *R* = 3. *P < 0.05; **P < 0.01. **(C)** Cleavage rate of parthenogenetic activated embryos derived from fresh (*N* = 199), aged (*N* = 216), and IMP-treated aged (*N* = 218) group. *R* = 4. Significant differences are represented by different capital letters (*P* < 0.01). **(D)** Representative images of parthenogenetic embryo development on day 7 derived from fresh, aged, and IMP-treated aged groups. Scale bar = 100 μm. **(E)** Blastocyst formation rate on day 7 in the fresh, aged, and IMP-treated aged groups. Significant differences are represented by different capital letters (*P* < 0.01). **(F)** Hatching rate on day 7 in the fresh, aged, and IMP-treated aged groups. No hatched blastocysts were observed in aged group. Significant differences are represented by different lower-case (*P* < 0.05) and capital (*P* < 0.01) letters.

As shown in Figure 1A, IMP supplementation helped oocytes reduce the cytoplasmic abnormalities, and spontaneous parthenogenetic activation. The expression levels of MOS proto-oncogene, serine/threonine kinase (MOS) in aged and IMP-treated aged oocytes were downregulated by 0.52 ± 0.15 and 0.76 ± 0.08-fold while the expression levels of cyclin B1 (CCNB1) in aged and IMP-treated aged oocytes were downregulated by 0.37 ± 0.03 and 0.78 ± 0.08-fold compared to the fresh oocytes. The expression levels of bone morphogenetic protein 15 (BMP15) in aged and IMP-treated aged oocytes were downregulated by 0.41 ± 0.11 and 0.74 ± 0.13-fold while the expression levels of growth differentiation factor 9 (GDF9) in aged and IMP-treated aged oocytes were downregulated by 0.38 ± 0.14 and 0.73 ± 0.14-fold compared to the fresh oocytes (Figure 1B). The cleavage rate in parthenogenetic embryos derived from fresh, aged, and IMP-treated aged oocytes were 85.71 ± 2.92%, 70.54 ± 5.89%, and 77.80 ± 5.67%, respectively (Figure 1C). The blastocyst formation rate in fresh, aged, and IMP-treated aged oocytes were 49.82 ± 2.67%, 16.27 ± 1.27%, and 30.97 ± 1.47% on day 7, respectively (Figures 1D,E). In addition, the hatching rates on day 7 in embryos derived from fresh and IMP-treated oocytes were 11.51 ± 1.16% and 8.22 ± 1.59%, respectively, while no hatched blastocyst was observed in aged oocytes (Figure 1F). These results suggested that IMP improved the quality of embryo derived from aged oocytes.

### IMP Supplementation Maintains Cortical Granule Distribution in Aged Oocytes

**FIGURE 2:**
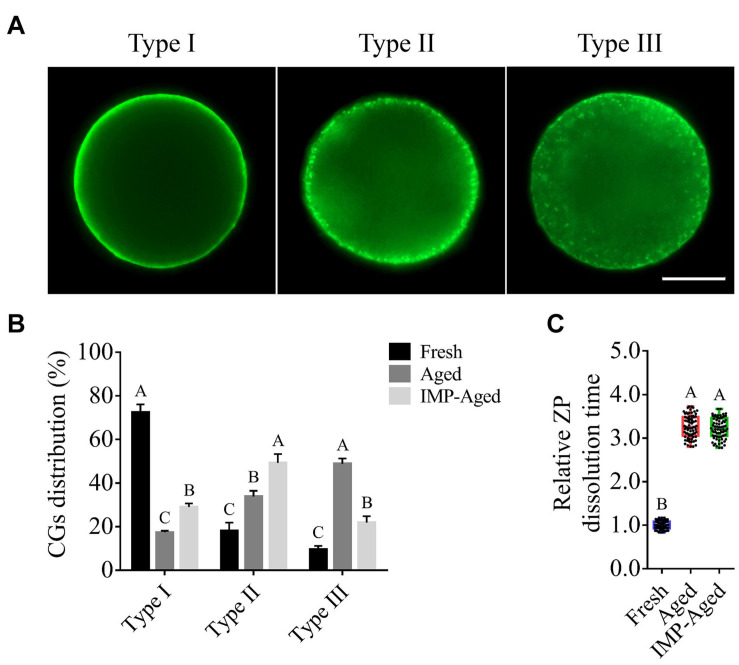
Effects of IMP on the distribution of cortical granule and ZP hardness in aged oocytes with/without IMP treatment. **(A)** Representative images of different types of cortical granule distribution. Scale bar = 25 μm. **(B)** The proportion of different types of oocytes in the fresh (*N* = 105), aged (*N* = 98), and IMP-treated aged groups (*N* = 100). *R* = 3. Significant differences are represented by different capital letters (*P* < 0.01). **(C)** Relative dissolution time of ZP in fresh (*N* = 75), aged (*N* = 75), and IMP-treated aged (*N* = 75) oocytes. No significant difference was observed in dissolution time of ZP between aged and IMP-treated aged oocytes, which suggested no significant difference in ZP hardness with or without IMP treatment. *R* = 3. Significant differences are represented by different capital letters (*P* < 0.01).

The process of oocyte aging is often accompanied by abnormal cortical granule distribution and changes in ZP hardness. In this study, the oocytes were classified into three types according to the observed distribution pattern of cortical granules (Figure 2A): Type 1: almost all cortical granules are distributed in the cortex and form a continuous halo around the plasma membrane; Type II: cortical granules are distributed in both the cytoplasm and plasma membrane, while halo formation around the plasma membrane is discontinuous; and Type III: cortical granules are more highly distributed in the cytoplasm than on the plasma membrane.

The percentages of Type I oocytes in fresh, aged, and IMP-treated aged group were 72.45 ± 3.62%, 17.33 ± 0.78%, and 28.96 ± 1.64%, respectively. The percentages of Type II oocytes in fresh, aged, and IMP-treated aged group were 18.02 ± 3.84%, 33.81 ± 2.65%, and 49.19 ± 4.11%, respectively. The percentages of Type III oocytes in fresh, aged, and IMP-treated aged group were 9.53 ± 1.64%, 48.86 ± 2.40%, and 21.86 ± 2.88%, respectively (Figure 2B). These results indicated that IMP supplementation maintained cortical granule distribution in aged oocytes. However, no significant difference in dissolution time of ZP was observed between aged and IMP-treated aged oocytes (*P* > 0.05; Figure 2C).

### IMP Supplementation Inhibited Apoptosis in Aged Oocytes

**FIGURE 3:**
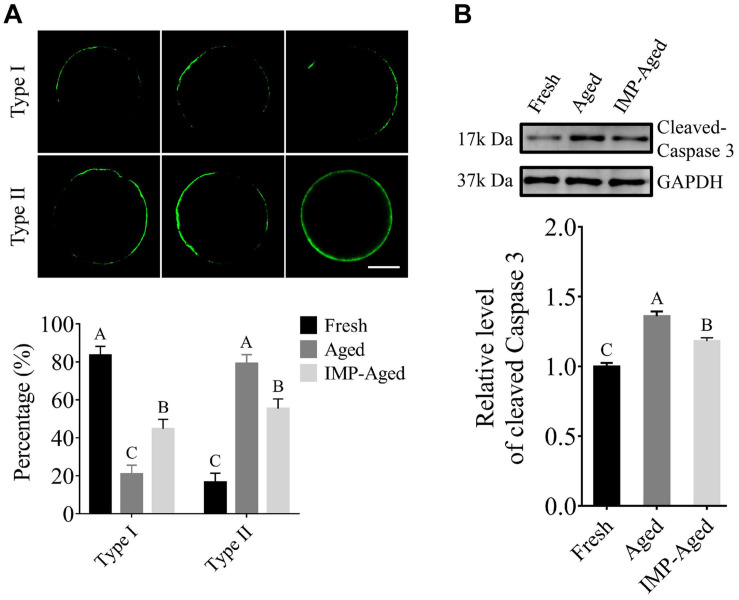
IMP treatment reduced apoptosis in aged oocytes. **(A)** Representative images of Annexin-V staining and proportion of Type I/II oocytes in fresh (*N* = 246), aged (*N* = 217), and IMP-treated aged (*N* = 235) groups. Scale bar = 25 μm. Significant differences are represented by different capital letters (*P* < 0.01). *R* = 3. **(B)** Cleaved-Caspase 3 level in fresh, aged, and IMP-treated aged groups. Significant differences are represented by different capital letters (*P* < 0.01). *R* = 3.

As shown in Figure 3A, In this study, the oocytes were classified into two types according to the observed distribution pattern of Annexin-V-positive membrane: Type 1: less than 50% of plasma membrane was labeled with Annexin-V-EGFP; Type II: more than 50% of plasma membrane was labeled with Annexin-V-EGFP. The percentages of Type I oocytes in fresh, aged, and IMP-treated aged group were 83.41 ± 4.76%, 20.84 ± 4.67%, and 44.64 ± 5.07%, respectively. The percentages of Type II oocytes in fresh, aged, and IMP-treated aged group were 16.59 ± 4.76%, 79.16 ± 4.67%, and 55.36 ± 5.07%, respectively. In addition, the level of cleaved-Caspase 3 in aged and IMP-aged oocytes were 1.36 ± 0.03 and 1.18 ± 0.02-times compared to those in fresh oocytes, respectively (Figure 3B).

### IMP Supplementation Enhanced the Oxidation Resistance of Aged Oocytes

**FIGURE 4:**
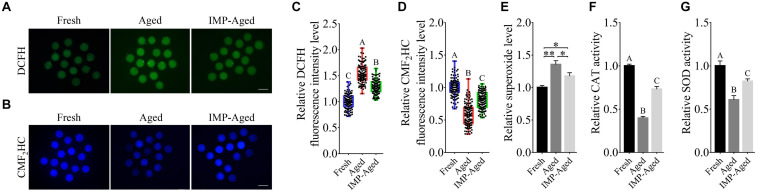
Effects of IMP on oxidation resistance in aged oocytes. **(A)** Representative DCFH staining images of oocytes in fresh, aged, and IMP-treated group. Scale bar = 100 μm. **(B)** Representative CMF_2_HC staining images of oocytes in fresh, aged, and IMP-treated group. Scale bar = 100 μm. **(C)** Relative DCFH fluorescence intensity (represents ROS) level in fresh (*N* = 120), aged (*N* = 123), and IMP-treated aged oocytes (*N* = 119). *R* = 3. Significant differences are represented by different capital letters (*P* < 0.01). **(D)** Relative CMF_2_HC fluorescence intensity (represents GSH) level in fresh (*N* = 127), aged (*N* = 124), and IMP-treated (*N* = 127) groups. *R* = 3. Significant differences are represented by different capital letters (*P* < 0.01). **(E)** Relative superoxide level change in fresh, aged, and IMP-treated aged groups. *R* = 3. *P < 0.05; **P < 0.01. **(F)** Relative CAT level change in fresh, aged, and IMP-treated aged groups. *R* = 3. Significant differences are represented by different capital letters (*P* < 0.01). **(G)** Relative SOD level change in fresh, aged, and IMP-treated aged groups. *R* = 3. Significant differences are represented by different capital letters (*P* < 0.01).

To determine the antioxidant effects of IMP on aged oocytes, ROS, and GSH levels were detected by DCFH and CMF_2_HC assays, respectively (Figures 4A,B). Results showed that the DCFH fluorescence intensity levels in aged and IMP-treated aged oocytes were significantly increased to 1.54 ± 0.17 and 1.29 ± 0.13-fold (Figure 4C), while the CMF_2_HC fluorescence intensity levels were significantly decreased to 0.58 ± 0.16 and 0.80 ± 0.13-times (Figure 4D) compared to those in fresh oocytes. The superoxide level in IMP-treated aged oocytes was lower than that in aged oocytes although it was still higher than those in the fresh oocytes (Figure 4E). In addition, IMP-treated aged oocytes also showed significantly higher activity of CAT (Figure 4F) and SOD (Figure 4G) than non-IMP-treated aged oocytes although their levels were still lower than those in the fresh oocytes. These results suggested that IMP reduces the accumulation of ROS and enhanced the antioxidant capacity in aged oocytes.

### IMP Supplementation Ameliorated Mitochondrial Dysfunction in Aged Oocytes

**FIGURE 5:**
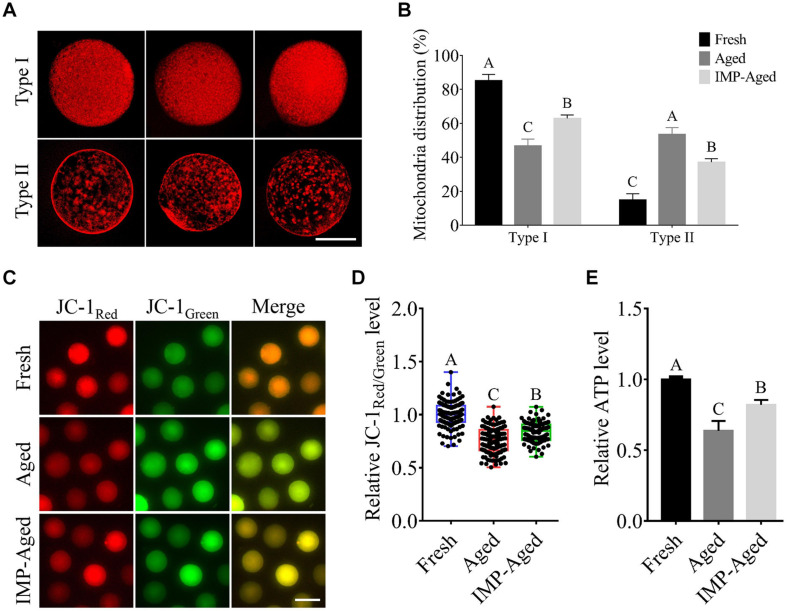
Effects of IMP on mitochondrial distribution, ΔΨm, and ATP levels in aged oocytes. **(A)** Representative images of Type I/II (three each) mitochondrial distribution in oocytes with MitoTracker staining. Scale bar = 50 μm. **(B)** The proportion of Type I/II oocytes in fresh (*N* = 119), aged (*N* = 76), and IMP-treated aged (*N* = 99) groups. Significant differences are represented by different capital letters (*P* < 0.01). *R* = 3. **(C)** Representative images of JC-1_*Red/Green*_ staining in fresh, aged, and IMP-treated aged oocytes. Scale bar = 100 μm. **(D)** Relative fluorescence intensity of JC-1_*Red/Green*_ in fresh (*N* = 128), aged (*N* = 122), and IMP-treated aged (*N* = 120) oocytes. Significant differences are represented by different capital letters (*P* < 0.01). *R* = 3. **(E)** Relative ATP levels in fresh, aged, and IMP-treated aged oocytes. *R* = 3. Significant differences are represented by different capital letters (*P* < 0.01).

The mitochondria are important for maintaining the quality of aged oocytes. In this study, the oocytes were classified into two types according to the observed mitochondrial distribution pattern: Type I: several mitochondria distributed evenly in the oocyte; and Type II: relatively fewer mitochondria with assembled distribution, while no mitochondria observed in certain places in the oocyte (Figure 5A).

Our results showed that percentage of Type I oocytes in the fresh, aged, and IMP-treated aged groups were 85.13 ± 3.66%, 46.61 ± 4.06%, and 62.84 ± 2.06%, respectively (*P* < 0.01), while the percentage of Type II oocytes were 14.87 ± 3.66%, 53.39 ± 4.06%, and 37.16 ± 2.06%, respectively (Figure 5B; *P* < 0.01).

Next, we measured the ΔΨm of aged oocytes by examining the ratio of red/green fluorescence using JC-1 dye staining. The results showed that ΔΨm in aged and IMP-treated aged oocytes were decreased to 0.76 ± 0.12 and 0.84 ± 0.09-fold compared to the fresh oocytes, respectively (Figures 5C,D).

Furthermore, the ATP levels were analyzed. The results showed that the ATP levels in aged and IMP-treated aged oocytes decreased by 0.63 ± 0.07 and 0.82 ± 0.03-fold compared to those in fresh oocytes, respectively (Figure 5E).

### IMP Supplementation Inhibited Autophagy in Aged Oocytes

**FIGURE 6:**
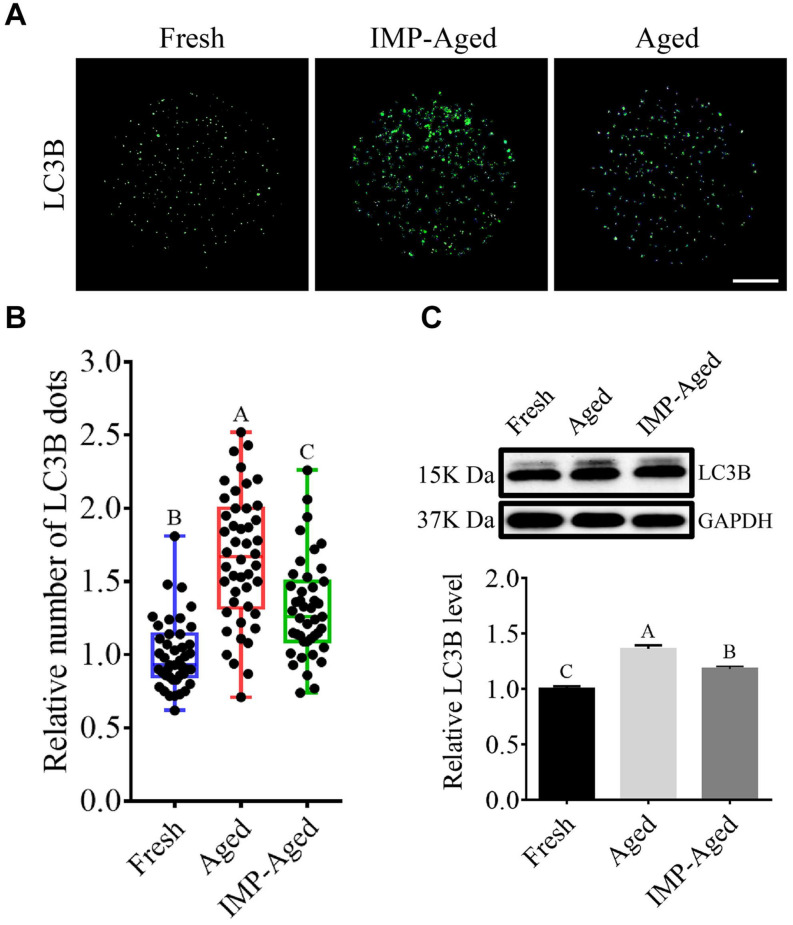
IMP supplementation reduced autophagy in aged oocytes. **(A)** Representative immunofluorescent images of LC3B dots in fresh, aged, and IMP-treated aged oocytes. Scale bar = 25 μm. **(B)** The relative number of LC3B dots in fresh (*N* = 43), aged (*N* = 44), and IMP-treated aged oocytes (*N* = 42). *R* = 4. **(C)** Expression of LC3B in fresh, aged, and IMP-treated aged oocytes. *R* = 3.

To assess whether IMP regulates autophagy in oocytes, LC3B levels were measured. As shown in Figures 6A,B, the relative number of LC3B dots in the aged and IMP-treated aged oocytes increased to 1.68 ± 0.43 and 1.32 ± 0.34-fold compared to that in fresh group, respectively. Western blotting results also showed that the level of LC3B in aged and IMP-treated aged oocytes increased to 1.36 ± 0.03 and 1.18 ± 0.02-fold compared to that in fresh oocytes, respectively (Figure 6C). These results suggested that autophagy levels were reduced with IMP supplementation.

### IMP Supplementation Ameliorated Impairment of Spindle/chromosome Structure in Aged Oocytes

**FIGURE 7:**
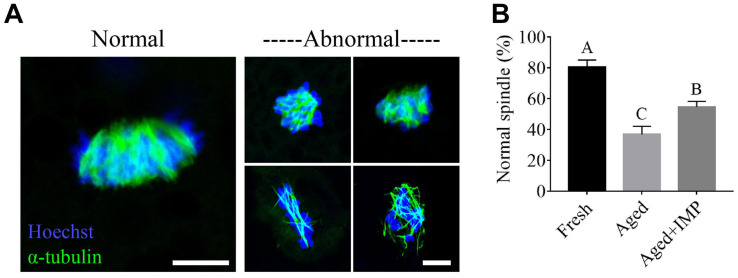
Effect of IMP supplementation on the spindle assembly in porcine aged oocytes. **(A)** Representative immunofluorescent images of normal/abnormal spindle morphologies and chromosome alignment in aged and IMP-treated aged porcine oocyte. Scale bar = 5 μm. **(B)** The rate of normal/abnormal spindle morphologies and chromosome alignment in fresh, aged, and IMP-treated aged porcine oocyte. Compared with aged oocytes (*N* = 114), the rate of normal spindle formation was significantly increased with IMP treatment (*N* = 115). However, it is still lower than that in the fresh oocytes (*N* = 127). Significant differences are represented with different capital letters (*P* < 0.01).

It has been known that oocyte aging is always caused defective spindle assembly, we thus further observed the organization of spindle/chromosome structure in fresh, aged, and IMP-treated oocytes. As shown in Figure 7A, most of fresh oocytes showed a normal barrel-shaped spindle apparatus with well-aligned chromosomes at the equatorial plate. However, various abnormal spindles and misaligned chromosomes were observed in aged oocytes. Quantitatively, the normal rates of spindle/chromosome structure in fresh, aged, and IMP-treated aged oocytes were 80.52 ± 4.59%, 36.84 ± 5.26%, and 54.65 ± 3.63%, respectively (Figure 7B).

## Discussion

**FIGURE 8:**
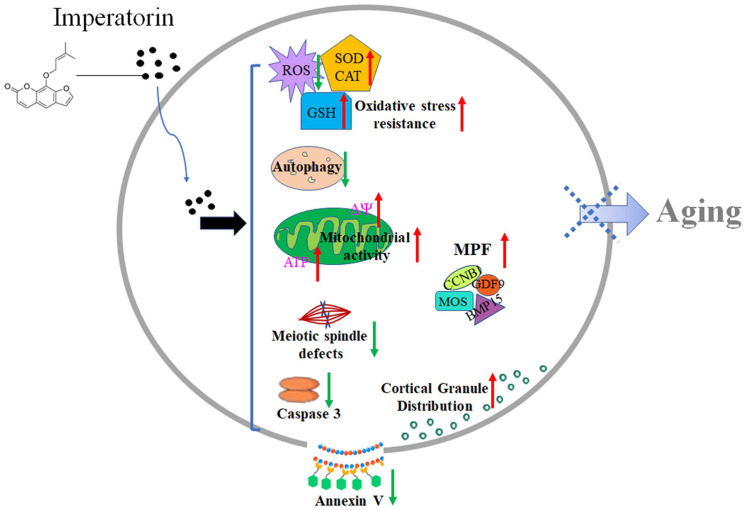
Schematic diagram of the effects of IMP on oocyte aging. After treatment of IMP, intracellular ROS, apoptosis, and autophagy levels were decreased, while activity of CAT and SOD as well as mitochondria function (ATP production) were increased in aged oocyte. IMP-treated aged oocytes also showed significantly higher levels of MPF (MOS, CCNB1, BMP15, and GDF9) and well cortical granule distribution. These may help oocyte delay aging process.

The aging of postovulatory oocytes has a significant negative impact on livestock production. Although the mechanism of aging is not yet fully clear, excessive accumulation of ROS is considered to be a major factor in inducing aging (Cui et al., 2011; Sasaki et al., 2019; Tamura et al., 2020). Here, our results demonstrated that IMP exhibited antioxidant effects and enhanced aged oocyte quality by reducing ROS accumulation, autophagy, and apoptosis, enhancing mitochondrial function, and subsequently promoting embryonic development (Figure 8).

In this study, we first confirmed that IMP improved the quality of aged oocytes, as well as the developmental competence of embryos derived from aged oocytes, especially the blastocyst formation and hatching rates. The changes in mRNA expression of related functional genes and relatively well-distributed cortical granules further supported these results. The up-regulated mRNA expression of GDF9, BMP15, MOS, and CCNB1 suggested that IMP might be involved in regulating oocyte cytoplasmic maturation (Su et al., 2004; De Castro et al., 2016; Liu et al., 2018), as well as promoting MPF activity (Peter et al., 2002; Madgwick and Jones, 2007; Yang et al., 2017). Moreover, stable MOS and CCNB1 levels are essential for meiosis mediated by MAD1, MPS1, and APC/C^*Cdc*20^ (Alfonso-Pérez et al., 2019), as well as balancing the MAPK, NF-κB signal which affects the ROS and mitochondrial dysfunction-mediated oocyte aging (Achache et al., 2020; Niu et al., 2020; Zhu et al., 2020). In addition, relatively high levels of GDF9 and BMP15 also help slow down the aging and reduce the apoptosis of oocytes by luteinizing hormone receptor, BCL2/BAX, connexin43-mediated regulating the steroidogenic acute regulatory protein expression, plasminogen activator, gap junctions (Hussein et al., 2005; Chang et al., 2014; De Castro et al., 2016). Simultaneously, we found that IMP supplementation also prevented the release of cortical granules, which is in accordance with previous findings that showed that postovulatory aging is highly correlated with a variety of defects in oocytes, including precocious release of cortical granules (Liu, 2011; Bianchi et al., 2015; Miao et al., 2018). However, no significant difference in ZP hardening was observed between IMP-treated and normal aged oocytes. This suggests that IMP may not be effective in improving the fertilization processing of aged oocytes.

Previous studies have shown that IMP regulates the activities of various antioxidant enzymes, including glutathione peroxidases, superoxide dismutase, glutathione reductase, thereby reducing the production of ROS (Raja et al., 2011; Sun et al., 2012; Cao et al., 2013). Here, our results showed that IMP treatment enhanced the level of GSH, activity of CAT and SOD in aged oocytes. As an intrinsic antioxidant present in oocytes, the content of GSH has been shown to decrease with the progression of oocyte aging and accumulation of ROS (Dumollard et al., 2007). In addition, relative higher levels of CAT and SOD would also enhance the antioxidant capacity of oocytes (Nie et al., 2020). Consistently, our results showed that the levels of ROS and superoxide were significantly decreased with IMP supplementation, which may be one of the reasons why ROS accumulation is relatively lower in IMP-treated aged oocytes. This also suggests that IMP treatment ameliorates ROS-induced oocyte aging during IVM (Agarwal et al., 2012).

The mitochondria are the principal producers of ATP for all energy-requiring cellular activities in oocytes, and are particularly vulnerable to ROS attack and functional damage in aged oocytes (Eichenlaub-Ritter et al., 2011). As previously reported, ATP production is reduced in aged oocytes (Koyama et al., 2014). However, here, IMP supplementation ameliorated low ATP production caused by aging-induced mitochondrial dysfunction. In addition, our study showed that MMP were significantly increased with IMP supplementation, indicating that IMP may help in maintaining high mitochondrial quality and ensuring the survival and viability of the offspring (Gemmell et al., 2004).

Spindles and actin filaments of oocyte are sensitive to chemicals and aging (Sun and Kim, 2012; Liang et al., 2018; Niu et al., 2020). Our results showed that the rate of abnormal spindle morphology in aged oocytes exposed to 40 μM IMP is markedly lower than that in control oocytes. This suggests that IMP reduces incidence of meiotic arrest and spindle abnormalities of aged oocytes *in vitro*, which may further reduce the failure of polar body extrusion from subcellular structure level (Wang et al., 2016). The mechanism may involve IMP acting as a regulator to affect the expressions of MAPK family and their phosphorylated levels (Cao et al., 2014; Li et al., 2015; Zhang et al., 2015). In addition, IMP may also alleviate the negative effects of aging on oocytes by regulating the Ca^2+^ from intracellular stores (He et al., 2007; Zhang et al., 2010). However, abnormal spindles and misaligned chromosomes were still observed in aged oocytes with IMP treatment, which means IMP cannot completely prevent spindle abnormalities during the aging process of oocytes.

During aging-induced mitochondrial dysfunction, the cells undergo significant changes in the levels of autophagy and apoptosis (Wang et al., 2009; Lin et al., 2018; Woods et al., 2018). Autophagy, a process by which cells degrade their cytoplasmic proteins or organelles to meet their metabolic needs, as well as self-renewal of organelles play an essential role in the process of oocyte maturation and aging (Mizushima, 2007). In our study, we observed that the LC3B protein was down-regulated with IMP supplementation. Consistent with previous studies, this result suggests that IMP helps in stabilizing the internal environment and reducing the autophagy of aging oocytes. Moreover, we also find that the level of cleaved-Caspase 3 was significantly decreased with IMP treatment, indicating that IMP exerts an anti-apoptotic effect via the caspase 3 pathway, which is consistent with previous studies (Wang et al., 2013; Nasser et al., 2019). In addition, this also suggests that IMP helps in overcoming ROS-induced survival pressure and inhibiting apoptosis and autophagy, as well as maintaining the quality of normal and aged oocytes (Fulda et al., 2010; Scherz-Shouval and Elazar, 2011; Marino et al., 2014).

Previous studies suggested that oxidative stress, which resulted from an imbalance between pro-oxidants and the body’s scavenging ability (antioxidants), influenced the entire reproductive lifespan of female animal and even thereafter (Agarwal et al., 2005). The health benefits of antioxidants-based diets could efficiently modulate oxidative and inflammatory stress to maintain the reproductive ability (Serafini and Peluso, 2016). Therefore, there is mounting interest in identifying foods, food extracts and phytochemical formulations. In this study, the *in vitro* research results showed that IMP had great potential on delaying the aging of oocyte. These are similar like Lycopene, vitamin C, coenzyme Q10, proanthocyanidin, which had been widely recognized for their anti-aging effects both *in vivo* and *in vitro* (Kaliora et al., 2006; Allemann and Baumann, 2008). This also indicates that IMP may have the potential to delay aging and improve animal reproduction as a food additive or therapeutic chemicals. However, the specific pathways that IMP affects in early embryos are unclear, and further research is needed.

## Conclusion

In summary, IMP supplementation improves the quality of aged porcine oocytes and subsequent embryonic development by increasing antioxidant capacity, reducing ROS accumulation, blocking premature exocytosis of cortical granules, preventing the abnormal mitochondria distribution, enhancing mitochondrial function, and inhibiting the occurrence of autophagy and apoptosis. These findings provide a theoretical basis for improvements of animal reproduction and *in vitro* embryo production.

## Data Availability Statement

The original contributions presented in the study are included in the article/Supplementary Material, further inquiries can be directed to the corresponding author/s.

## Ethics Statement

The animal study was reviewed and approved by the Experimental Animal Center, Institutional Animal Care and Use Committee of Jilin University (IACUC-ID-201802070).

## Author Contributions

J-bZ, N-HK, and HJ conceived and designed the experiments. DL, S-pL, WL, and X-rY performed the experiments. DL and X-rY analyzed the data. DL wrote the manuscript. DL, WL, HG, and Z-lJ generated data handling, image analysis, and visualization tools. J-bZ, N-HK, HJ, Y-xJ, and BY gave general advice and edited the manuscript. All authors contributed to the article and approved the submitted version.

## Conflict of Interest

The authors declare that the research was conducted in the absence of any commercial or financial relationships that could be construed as a potential conflict of interest.
