# Supplementary material for: Imperatorin Ameliorates the Aging-Associated Porcine Oocyte Meiotic Spindle Defects by Reducing Oxidative Stress and Protecting Mitochondrial Function
Source: Front Cell Dev Biol. 2020 Dec 21;8:592433. doi: 10.3389/fcell.2020.592433 (PMC7779485; doi:10.3389/fcell.2020.592433)
Supplement: Supplementary file 1 [file Table_1.DOCX]

Supplementary Material

**Supplementary Table S1. Primer sequences used for qRT-PCR analysis**

| Genes | Sequences 5’-3’ | Product size (bp) | Accession number |
| --- | --- | --- | --- |
| GAPDH | F: GGGCGTGAACCATGAGAAGT | 230 | NM_001206359.1 |
|  | R: AAGCAGGGATGATGTTCTGG |  |  |
| MOS | F: TGGGAAGAAACTGGAGGACA | 121 | NM_001113219.1 |
|  | R: TTCGGGTCAGCCCAGTTCA |  |  |
| CCNB1 | F: CCAACTGGTTGGTGTCACTG | 195 | NM_001170768.1 |
|  | R: GCTCTCCGAAGAAAATGCAG |  |  |
| BMP15 | F: CCCTCGGGTACTACACTATG | 192 | AF458070.2 |
|  | R: GGCTGGGCAATCATATCC |  |  |
| GDF9 | F: GAGCTCAGGACACTGTAAGCT | 272 | NM_001001909.1 |
|  | R: CTTCTCGTGGATGATGTTCTG |  |  |

F: forward primer; R: reverse primer. Annealing temperature = 60 ℃
